# Modulation of Lower Limb Biomechanics and Muscle Activation by Midsole Hardness in Children During Running

**DOI:** 10.1002/jfa2.70211

**Published:** 2026-09-08

**Authors:** Zhaolong Ye, Bojie Xuan, Fengping Li, Kuiyu Chen, Yiyao Chen, Zhanyi Zhou, Diwei Chen, Dongxu Wang, Xuanzhen Cen, Yufei Li, Dong Sun, Gusztáv Fekete, Danica Janicijevic, Yang Song, Yaodong Gu

**Affiliations:** ^1^ Faculty of Sports Science Ningbo University Ningbo China; ^2^ Research Academy of Grand Health Ningbo University Ningbo China; ^3^ ANTA Kids Anta Sports Products Limited Xiamen China; ^4^ Department of Materials Science and Mechanical Engineering AUDI Hungaria Faculty of Engineering Széchenyi István University Győr Hungary; ^5^ Department of Sports Sciences and Physical Conditioning Faculty of Education Universidad Católica de la Santísima Concepción Concepción Chile; ^6^ Department of Biomedical Engineering The Hong Kong Polytechnic University Hong Kong China; ^7^ Research Institute of Sport Science Hungarian University of Sports Science Budapest Hungary

**Keywords:** iEMG, lower‐extremity biomechanics, midsole hardness, navicular height, pediatric running

## Abstract

**Introduction:**

Children's developing musculoskeletal systems are highly sensitive to mechanical stimuli. This study investigated the effects of running shoe midsole hardness (MH) (soft midsole [SM], medium midsole [MM], and hard midsole [HM]) on lower‐limb biomechanics and muscle activation during the running stance phase in children.

**Methods:**

Thirty healthy boys participated. Kinematic, kinetic, electromyographic, and navicular height (NH) data were collected during running. Differences across the three hardness conditions were analyzed using one‐way repeated‐measures ANOVA.

**Results:**

Maximum ankle sagittal angle decreased from 29.12° (SM) to 27.11° (HM) (*p* = 0.008), and hip sagittal range of motion (ROM) decreased from 43.06° (SM) to 39.66° (HM) (*p* = 0.047). NH was significantly higher under MM (12.48 mm) and HM (12.93 mm) than under SM (10.92 mm) (both *p* < 0.001). Vertical average loading rate (VALR) was significantly higher in HM (64.18 BW/s) than SM (53.14 BW/s; *p* = 0.032). Regarding muscle activation, significant main effects of MH were observed for the relative contributions of the tibialis anterior (TA), lateral gastrocnemius (LG), and semitendinosus (ST) (*p* = 0.046, *p* = 0.010, and *p* = 0.011, respectively). Post hoc comparisons showed that TA and LG contributions were lower under HM than under SM, whereas ST contribution was higher under HM than under SM and MM. Gluteus maximus (GM) activation (0.38) and relative contribution (17.5%) were highest under MM, with a significant main effect of MH (*p* = 0.002).

**Conclusions:**

Midsole hardness altered selected lower‐limb kinematics, navicular height, vertical loading rate, and muscle activation during running in children; however, these acute differences do not indicate functional superiority of any of the tested hardness conditions.

## 1 Introduction

Running is a fundamental component of physical activity in children and exposes the developing musculoskeletal system to repeated impact forces during ground contact [1, 2]. As the direct interface between the foot and the ground, running footwear can modify the mechanical conditions experienced by the lower extremities [3]. Among footwear properties, midsole hardness (MH) is particularly relevant because it influences midsole deformation and the mechanical interaction between the foot and the ground during running. However, much of the existing evidence regarding the biomechanical effects of MH has been obtained from adults, whereas corresponding evidence in children remains limited [4, 5]. Consequently, the effects of MH on lower‐limb kinematics, impact characteristics, and muscle activation during running in children remain insufficiently understood.

Previous studies have shown that changes in midsole mechanical properties can affect vertical impact characteristics and lower‐limb biomechanical responses during running [5, 6]. Softer midsoles generally allow greater material deformation and have been associated with lower vertical average loading rates (VALR) during ground contact [7]. Conversely, harder footwear conditions permit less material deformation and may be associated with higher VALR [8, 9, 10]. Hoffman et al. (2015) showed that footwear condition can influence dynamic navicular motion during running, suggesting that changes at the shoe–foot interface may affect medial longitudinal arch behavior [11, 12]. Together, these findings indicate that footwear mechanical properties may influence VALR, foot‐arch motion, and lower‐limb movement; however, these responses have rarely been examined concurrently in children.

Children should not simply be regarded as smaller adults when interpreting footwear‐related biomechanical responses. School‐age children within the age range examined in the present study (7–12 years) are still undergoing musculoskeletal and neuromotor development [13, 14, 15]. These developmental characteristics suggest that findings obtained from adults may not be directly generalizable to children. Previous pediatric footwear studies have primarily examined factors such as shoe flexibility and general footwear conditions, whereas relatively few studies have isolated MH while keeping other footwear characteristics as consistent as possible [2, 16]. Meanwhile, perceived comfort remains an important consideration in children's footwear selection, whereas biomechanical evidence concerning specific mechanical properties such as MH remains limited [17]. Consequently, the effects of systematic changes in MH on children's running biomechanics are still not well understood.

To evaluate the biomechanical effects of footwear interventions, previous studies have frequently focused on the ankle–foot complex, which directly interacts with the ground during foot contact [18]. Owing to its multi‐planar mobility, the ankle–foot complex can respond directly to changes in the mechanical properties of footwear and plays an important role in lower‐limb movement during running [18, 19]. However, changes at the foot–shoe interface may also be accompanied by changes in knee and hip motion and in the activation of lower‐limb muscles [20, 21]. Therefore, observations confined to the ankle–foot complex may not fully characterize the responses of the lower limb to changes in MH [22]. Examining the foot, ankle, knee, and hip together may provide a more comprehensive description of multi‐joint responses to different midsole‐hardness conditions [23, 24].

To characterize these responses from complementary biomechanical perspectives, this study assessed NH together with lower‐limb joint kinematics, VALR, and surface electromyography (sEMG). NH was used to characterize medial longitudinal arch behavior during stance, whereas joint kinematics described changes in lower‐limb movement across the ankle, knee, and hip. VALR was included to characterize vertical impact characteristics during early stance, and sEMG was used to assess the activation and relative contribution of selected lower‐limb muscles under different MH conditions [25, 26, 27]. Examining these outcomes within the same experimental protocol enables a multidimensional assessment of foot‐arch behavior, multi‐joint kinematics, vertical impact characteristics, and muscle activation, thereby providing a more comprehensive description of the acute biomechanical responses of children to different midsole‐hardness conditions.

Accordingly, this study aimed to examine the effects of three running‐shoe midsole‐hardness conditions on lower‐limb joint kinematics, NH, vertical impact loading, and the activation and relative contribution of selected lower‐limb muscles in healthy boys during running. Based on previous observations that softer midsoles permit greater material deformation and may reduce loading rate, whereas harder footwear conditions may restrict foot‐arch deformation, we hypothesized that increasing MH would be associated with higher VALR and NH and reduced sagittal‐plane lower‐limb motion [7, 8, 11]. We further hypothesized that changes in MH would alter the activation and relative contribution of selected lower‐limb muscles [20]. By characterizing these acute responses, the present study may provide preliminary biomechanical evidence to inform future research on children's running footwear.

## 2 Methods

### 2.1 Study Participant Details

An a priori power analysis was conducted utilizing G*Power 3.1 software (Franz Faul, Germany) to determine the requisite sample size for a one‐way repeated‐measures analysis of variance (ANOVA). Based on parameter settings adopted in a previous repeated‐measures biomechanics study, a conventional medium effect size of *f* = 0.25, an alpha level of *α* = 0.05, and a statistical power of 0.80 were specified [28]. The computation demonstrated that a minimum of 28 participants was required. Subsequently, to account for potential data invalidity or participant attrition during the experimental phase, 30 healthy male children (age: 9.75 ± 1.71 years; height: 142.12 ± 15.11 cm; body mass: 35.03 ± 11.51 kg; uniformly right‐leg dominant) were ultimately enrolled. Only male participants were included to maintain a homogeneous study sample and to avoid introducing sex as an additional source of between‐participant variability. In children of a similar age, sex‐related differences in running biomechanics appear to vary across biomechanical outcomes. Previous studies have reported no significant sex differences in spatiotemporal running parameters, including cadence and step length, or in foot‐strike pattern and foot rotation [1, 29]. However, some sex‐related differences in pelvic and hip kinematics may already be present before puberty and become more pronounced with maturation [30]. Leg dominance was determined by asking each participant which leg they would preferentially use to kick a ball, and all participants identified the right leg as their dominant leg [31]. Furthermore, strict eligibility criteria were established for participant selection, necessitating (1) normal plantar morphology, objectively confirmed via three‐dimensional (3D) foot scanning, thereby excluding individuals presenting with pes cavus (high arches) or pes planus (flat feet); (2) an absence of any lower‐extremity musculoskeletal injuries within the preceding 6 months; and (3) strict abstinence from strenuous physical exertion for 48 h prior to the testing protocol. Notably, the experimental methodology strictly complied with the ethical tenets of the Declaration of Helsinki and was officially approved by the Scientific Ethics Committee of Ningbo University (Approval No.: TY2025065). Before the initiation of formal experiments, the research objectives, testing procedures, and potential risks were comprehensively elucidated to all participants and their respective legal guardians, from whom written informed consent was subsequently obtained.

### 2.2 Experimental Protocol

**FIGURE 1:**
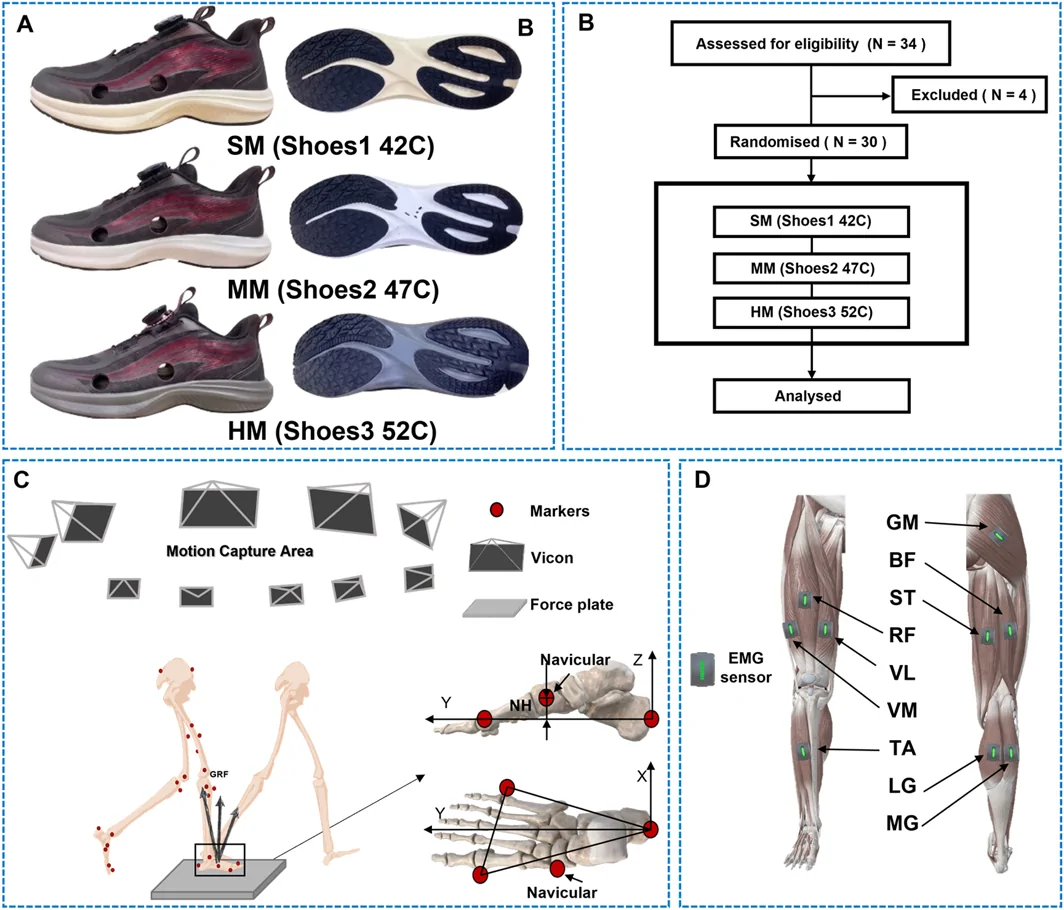
Experimental workflow and instrumental setup for biomechanical and neuromuscular data acquisition. (A) Test shoes; (B) experimental protocol; (C) experimental setup; (D) EMG marker placement location.

All experimental trials were executed within the environmentally controlled Biomechanics Laboratory at Ningbo University. Before data acquisition, a preliminary foot morphology screening was conducted utilizing an infrared foot scanner (efoot‐350Pros, 3DOE Technology Co. Ltd., Shenzhen) [32]. This procedure explicitly excluded individuals presenting with pes planus (flat feet) or pes cavus (high arches), thereby verifying normal plantar architectures across the cohort. The utilized methodology employed customized running footwear of an identical model (ANTA), engineered with three distinct MH configurations: soft midsole (SM, 42 Shore C), medium midsole (MM, 47 Shore C), and hard midsole (HM, 52 Shore C) (Figure 1A). The terms “soft,” “medium,” and “hard” were used as relative descriptors of the three experimental conditions, corresponding to the lowest, intermediate, and highest hardness levels tested in the present study. To guarantee methodological rigor, the hardness of each shoe was quantitatively assessed and calibrated via a Shore C durometer, ensuring that all structural and design parameters remained strictly consistent, with the sole independent variable being MH. Subsequently, following the shoe aperture assessment protocol delineated by Bishop et al., circular fenestrations (25 mm in diameter) were meticulously excised at predefined anatomical landmarks on the instep, medial, and lateral aspects of the shoe upper. This modification permitted unencumbered multi‐planar displacement of the surface markers during in‐shoe gait analysis while simultaneously preserving the structural integrity of the upper [33]. The main experimental procedure is illustrated in the schematic (Figure 1B).

During the testing phase, participants performed an overground running task at a target speed of 2.5 m/s along a level indoor runway, with the force plate embedded flush with the surrounding floor (Figure 1B). Running speed was monitored using photoelectric timing gates, and trials were considered valid when the measured speed was within ± 5% of the target speed. Before formal data collection, participants performed several practice runs to become familiar with the running task. Experimental trials were completed under all three footwear conditions, with the sequence randomized for each participant to mitigate potential order effects. Five valid running trials were recorded per condition [34]. In addition to meeting the prescribed speed criterion, a valid trial required complete right‐foot contact with the force plate without obvious gait adjustment and successful recording of the entire stance phase. A 1‐min rest interval was provided between consecutive trials to minimize potential fatigue. The outcome variables from the five valid trials were averaged for each participant within each condition before statistical analysis.

### 2.3 Data Collection

To capture the kinematic and kinetic variables of the lower extremities, a three‐dimensional (3D) motion capture system equipped with eight cameras (Vicon, Oxford Metrics Ltd., Oxford, UK) operating at a sampling frequency of 200 Hz was synchronized with a Kistler 3D force plate (Kistler Instrumente AG, Winterthur, Switzerland) sampling at 2000 Hz (Figure 1C) [35]. Subsequently, a total of 40 reflective markers were affixed to predefined anatomical bony landmarks on the subjects. These markers were utilized to construct an individualized musculoskeletal model based on the OpenSim Gait2392 architecture [36, 37]. Notably, cranial markers inherent to the default model were omitted; instead, two supplementary markers were positioned bilaterally on the navicular bones. This modification was implemented to enhance the precision of foot‐specific kinematic and kinetic data acquisition, thereby yielding a highly customized lower‐extremity biomechanical model.

Furthermore, sEMG signals were acquired in strict compliance with the SENIAM guidelines concerning anatomical landmark identification. Bipolar surface electrodes of the Delsys Trigno wireless sEMG system (Delsys, Boston, USA) were systematically positioned over nine target muscles: the gluteus maximus (GM), biceps femoris (BF), semitendinosus (ST), rectus femoris (RF), vastus lateralis (VL), vastus medialis (VM), tibialis anterior (TA), medial gastrocnemius (MG), and lateral gastrocnemius (LG) (Figure 1D) [38]. The electrodes were aligned precisely parallel to the orientation of the underlying muscle fibers. Before electrode application, the targeted epidermal areas were shaved and rigorously cleansed with 75% isopropyl alcohol swabs to minimize skin impedance. The captured sEMG signals were digitized at a sampling frequency of 1000 Hz. Ultimately, all electrophysiological data were recorded and archived in real‐time via the EMGworks software suite (Delsys, Boston, USA) to facilitate subsequent analytical processing.

### 2.4 Data Processing

The acquired experimental data were processed utilizing Vicon Nexus 2.6.1 software. This procedural step encompassed marker tracking, identification, trajectory gap interpolation, and anatomical labeling. Furthermore, stance phase intervals were delineated by extracting force plate data corresponding to a vertical reaction force threshold exceeding 20 N, thereby identifying the stance phase from right‐foot initial contact (IC) to toe‐off [39]. Subsequently, following exportation in the c3d format, the datasets were converted into .trc files via MATLAB R2022a and imported into the OpenSim environment. The musculoskeletal model was proportionally scaled to each participant's height and body mass, after which the hip, knee, and ankle joint angles were computed employing inverse kinematics (IK) [40]. The kinematic trajectories were smoothed utilizing a fourth‐order low‐pass filter with a 10‐Hz cutoff frequency. Notably, previous biomechanical investigations have demonstrated that applying an excessively low cutoff frequency artificially attenuates impact peaks, thereby underestimating the actual VALR [41]. Consequently, to preserve the high‐frequency fidelity of the impact transients, the vertical ground reaction force (vGRF) data were distinctly filtered using a fourth‐order low‐pass filter with a higher cutoff frequency of 50 Hz.

Medial longitudinal arch behavior was characterized using NH [25]. Specifically, a local reference plane was defined by three reflective markers positioned on the posterior aspect of the calcaneus and the distal ends of the first and fifth metatarsals. An additional marker was positioned on the medial aspect of the navicular bone (Figure 1C). NH was calculated as the perpendicular distance from the navicular marker to this local reference plane at each frame during stance, and the minimum value during the stance phase was extracted for analysis. A greater NH indicated a higher navicular position relative to the reference plane. NH was expressed in millimeters and was not normalized to foot length. The minimum NH values from the five valid trials were averaged for each participant within each footwear condition [42].

Initially, the raw sEMG signals were subjected to a bandpass filter operating between 20 and 450 Hz to eliminate motion artifacts and extraneous environmental noise, thereby preserving signal fidelity. Subsequently, the filtered data were full‐wave rectified—converting all negative amplitudes to absolute positive values—to prepare the dataset for downstream analysis. These rectified signals were then further smoothed utilizing a fourth‐order Butterworth low‐pass filter with a 20‐Hz cutoff frequency, yielding a linear envelope indicative of overarching neuromuscular activation. Notably, owing to the inherent physiological and cognitive challenges pediatric participants may encounter when performing a stable maximum voluntary isometric contraction (MVIC), a dynamic normalization approach was adopted. For each participant and each muscle, the sEMG signals were normalized to the maximum activation amplitude recorded across all valid trials under the three footwear conditions [43]. During the subsequent analytical phase, the integrated electromyography (iEMG) for each trial was computed via numerical integration of the normalized linear envelope over the stance phase (quantifying the area under the curve), which furnished a comprehensive measure of neuromuscular activation [44, 45]. The relative contribution of each muscle was calculated as the iEMG of that muscle divided by the summed iEMG of all nine measured muscles and expressed as a percentage, such that the relative contributions of the nine muscles summed to 100% for each participant under each experimental condition [46]. Average muscle activation was calculated as the mean amplitude of the normalized linear envelope during the stance phase.

VALR was employed to assess the landing impact during the stance phase of running [26]. Specifically, this metric was computed by deriving the average slope of the secant line intersecting the 20% and 80% thresholds of the vertical impact peak within the filtered vGRF data; this distinct interval was selected as it constitutes the most highly linear segment of the initial loading curve [47]. Consequently, the derivation was executed based on the Fz curve utilizing the following equation:

$$\text{VALR}=\frac{\Delta F}{\Delta t}=\frac{F_{80\%}-F_{20\%}}{t_{80\%}-t_{20\%}}$$

where ΔF denotes the force interval ranging from 20% to 80% of the peak vertical reaction force, and Δt represents the corresponding temporal duration.

### 2.5 Statistical Analysis

To evaluate the variations in biomechanical parameters across the three distinct MH conditions (SM, MM, and HM), a one‐way repeated‐measures analysis of variance (ANOVA) was conducted. Before the primary inferential testing, data normality was assessed using the Shapiro–Wilk test, and the assumption of sphericity was evaluated using Mauchly's test. When the assumption of sphericity was violated, the Greenhouse–Geisser correction was applied. Subsequently, upon the detection of a statistically significant main effect, post hoc pairwise comparisons were executed. A Bonferroni correction was applied to these comparisons to control for multiple testing. The alpha level for statistical significance was established a priori at *α* = 0.05, and the magnitude of the observed effects was quantified using partial eta squared (*ηp* ^2^). All descriptive statistics are expressed as mean ± SD.

## 3 Results

### 3.1 Lower‐Limb Joint Kinematics

**FIGURE 2:**
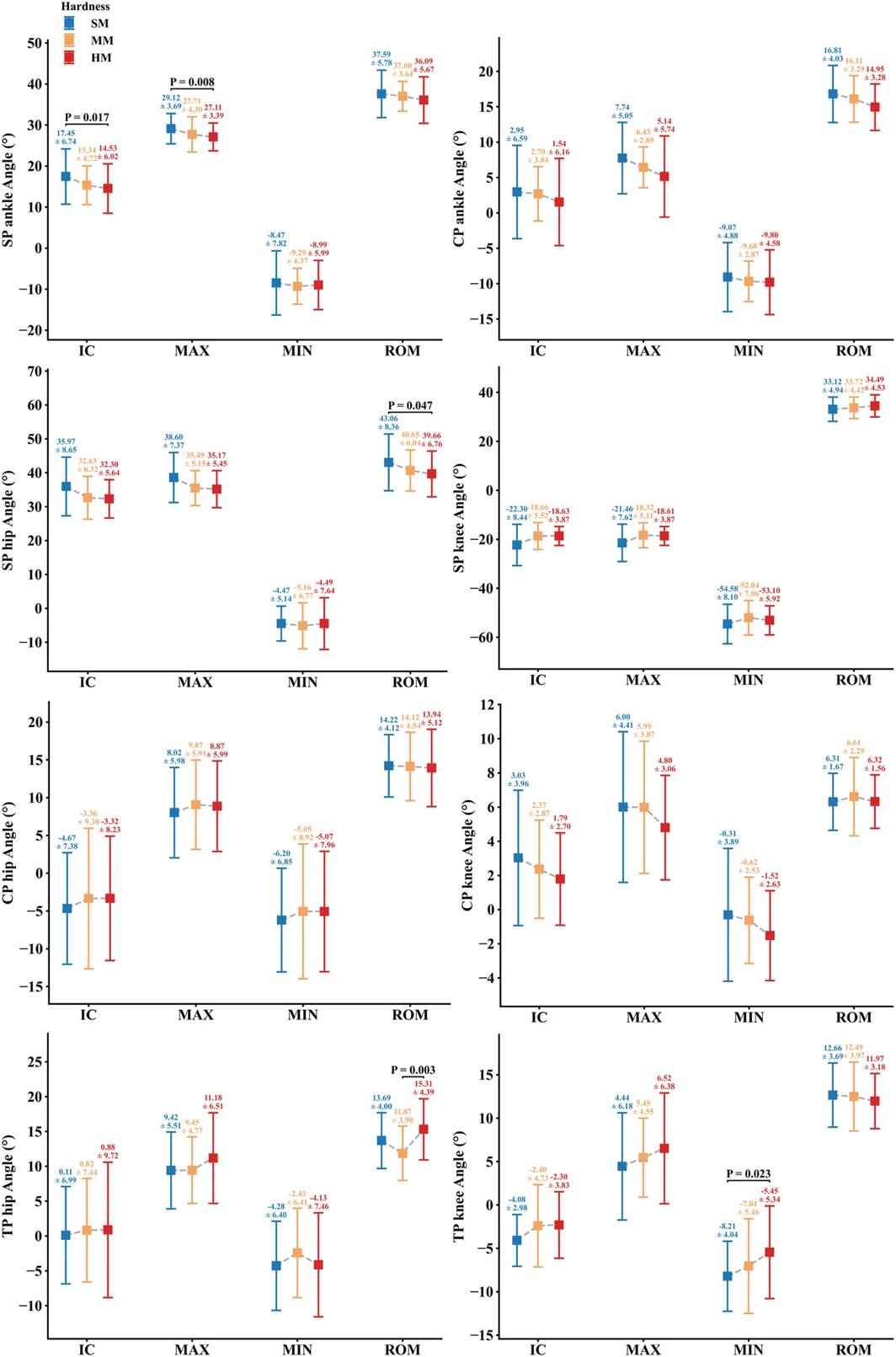
Comparative analysis of lower limb joint kinematics under different hardness conditions. CP: coronal plane; HM: high midsole (52C); IC: initial contact; MM: medium midsole (47C); ROM: range of motion; SM: soft midsole (42C); SP: sagittal plane; TP: transverse plane. Bold *p*‐values indicate a statistically significant difference (*p* < 0.05).

MH had a significant main effect on both the maximum sagittal angle of the ankle joint (*F* = 3.323, *p =* 0.043, $\eta_{p}^{2}$ = 0.106) and the angle at IC (*F* = 4.168, *p =* 0.021, $\eta_{p}^{2}$ = 0.130) (Figure 2). Bonferroni post hoc tests indicated that the maximum sagittal angle under the SM condition (29.12°) was significantly greater than that under the HM condition (27.11°, *p* = 0.008), whereas the MM condition showed no significant difference from the other two conditions. At the same time, the angle at IC under the SM condition (17.45°) was significantly greater than that under the HM condition (14.53°, *p =* 0.017).

In the sagittal plane of the knee joint, there were significant main effects for both angle at IC (*F* = 4.076, *p =* 0.031, $\eta_{p}^{2}$ = 0.127) and minimum flexion angle (*F* = 3.377, *p =* 0.041, $\eta_{p}^{2}$ = 0.108) across different MH conditions; however, post hoc tests did not reveal any statistically significant differences between groups. In the transverse plane, the main effect of the minimum rotation angle of the knee joint was significant (*F* = 3.732, *p =* 0.030, $\eta_{p}^{2}$ = 0.118); however, no significant pairwise differences were identified following Bonferroni correction.

MH significantly altered the sagittal plane angle at IC (*F* = 3.316, *p =* 0.042, $\eta_{p}^{2}$ = 0.084) and maximum flexion angle (*F* = 3.721, *p =* 0.038, $\eta_{p}^{2}$ = 0.094) of the hip joint. Additionally, the sagittal plane ROM of the hip joint also showed a significant effect (*F* = 5.104, *p =* 0.016, $\eta_{p}^{2}$ = 0.124). Post hoc tests further confirmed that the ROM of the hip joint under the SM condition (43.06°) was significantly greater than that under the HM condition (39.66°, *p =* 0.047).

### 3.2 Height of the Navicular Bone

**FIGURE 3:**
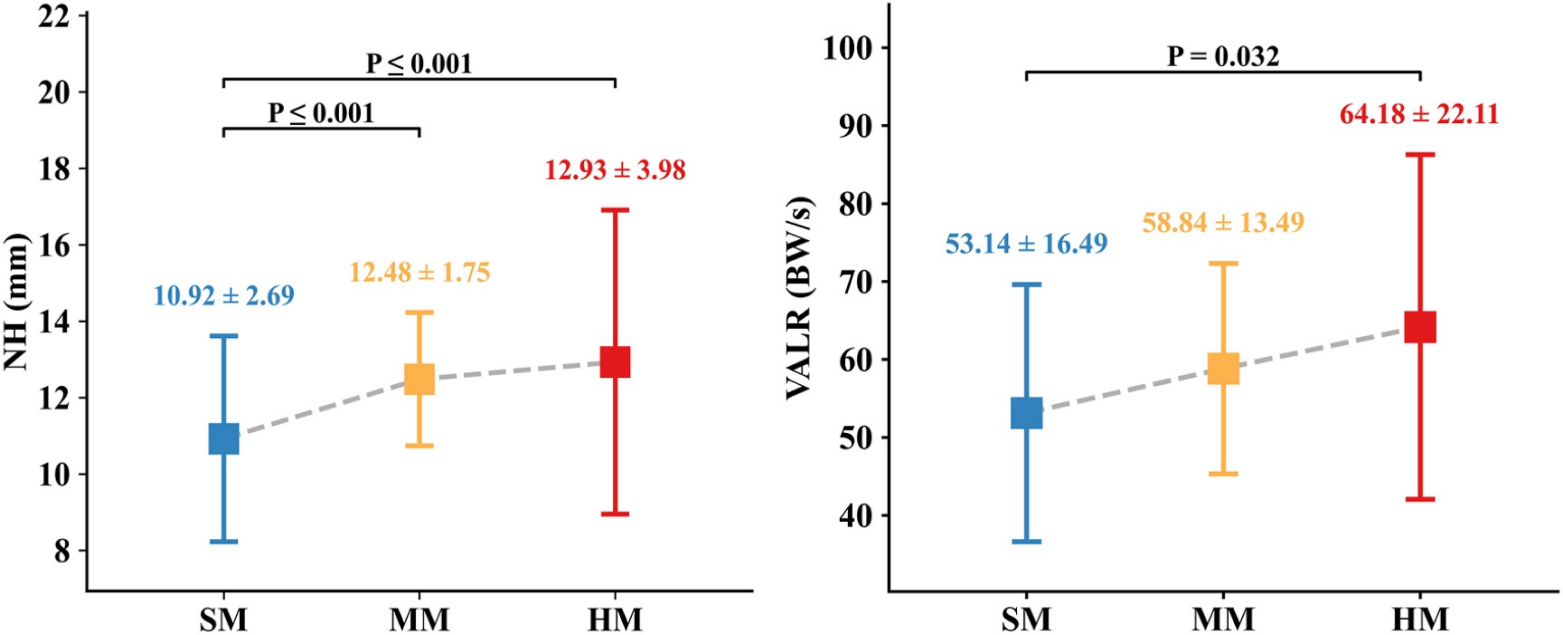
Effects of midsole hardness on NH and VALR during the running stance phase. HM: high midsole (52C); MM: medium midsole (47C); NH: navicular height; SM: soft midsole (42C); VALR: vertical average loading rate.

Different MH conditions produced a significant main effect on the height of the navicular bone during the subjects' running stance phase (*F* = 23.653, *p <* 0.001, $\eta_{p}^{2}$ = 0.449) (Figure 3A). Post hoc pairwise comparisons further revealed the specific sources of the differences in the data. The NH was lowest under the SM (10.92 mm) condition and was significantly lower than under the MM (12.48 mm, *p <* 0.001) and HM (12.93 mm, *p <* 0.001) conditions.

### 3.3 Vertical Average Loading Rate

The effects of different MH on VALR are shown in Table 1. The results of the repeated measures ANOVA indicate a significant main effect of MH on VALR (*F* = 3.775, *p =* 0.038, $\eta_{p}^{2}$ = 0.095) (Figure 3B). Bonferroni post hoc tests indicated that VALR was highest under the HM condition (64.18 BW/s) and was significantly higher than that under the SM condition (53.14 BW/s, *p* = 0.032). Although the VALR under the MM condition(58.84 BW/s) was higher than that under the SM condition, the difference did not reach statistical significance (*p >* 0.05), and there was also no significant difference between the MM and HM conditions (*p >* 0.05).

**TABLE 1:** Comparison of children's lower limb biomechanics during running across different midsole hardness conditions.

Subject			LS mean (SD)	MS mean (SD)	HS mean (SD)	*F*	*p*	$\eta_{p}^{2}$
Hip	SP	IC	35.97 (8.65)	32.63 (6.32)	32.30 (5.64)	3.316	**0.042**	0.084
		MAX	38.60 (7.37)	35.49 (5.15)	35.17 (5.45)	3.721	**0.038**	0.094
		MIN	−4.46 (5.14)	−5.16 (6.77)	−4.49 (7.64)	0.238	0.789	0.007
		ROM	43.06 (8.36)	40.65 (6.04)	39.66 (6.76)	5.104	**0.016**	0.124
	CP	IC	−4.67 (7.38)	−3.35 (9.30)	−3.32 (8.23)	0.532	0.59	0.013
		MAX	8.02 (5.98)	9.07 (5.91)	8.87 (5.99)	0.697	0.501	0.017
		MIN	−6.20 (6.85)	−5.05 (8.92)	−5.07 (7.96)	0.436	0.618	0.011
		ROM	14.22 (4.12)	14.12 (4.54)	13.94 (5.12)	0.057	0.944	0.001
	TP	IC	0.12 (6.99)	0.82 (7.44)	0.88 (9.72)	0.169	0.804	0.006
		MAX	9.42 (5.51)	9.45 (4.77)	11.18 (6.51)	1.397	0.254	0.046
		MIN	−4.28 (6.40)	−2.43 (6.41)	−4.13 (7.46)	0.995	0.363	0.033
		ROM	13.69 (4.00)	11.87 (3.90)	15.31 (4.39)	6.923	**0.002**	0.193
Knee	SP	IC	−22.30 (8.44)	−18.66 (5.52)	−18.63 (3.87)	4.076	**0.031**	0.127
		MAX	−21.46 (7.62)	−18.32 (5.11)	−18.61 (3.87)	3.377	**0.041**	0.108
		MIN	−54.58 (8.10)	−52.04 (7.06)	−53.10 (5.92)	1.663	0.199	0.056
		ROM	33.12 (4.94)	33.72 (4.42)	34.49 (4.53)	0.8	0.454	0.028
	CP	IC	3.03 (3.96)	2.37 (2.87)	1.79 (2.70)	1.683	0.195	0.057
		MAX	6.00 (4.41)	5.99 (3.87)	4.80 (3.06)	1.165	0.319	0.04
		MIN	−0.31 (3.89)	−0.62 (2.52)	−1.52 (2.63)	1.578	0.215	0.053
		ROM	6.31 (1.67)	6.61 (2.29)	6.32 (1.56)	0.254	0.777	0.009
	TP	IC	−4.08 (2.98)	−2.40 (4.73)	−2.30 (3.83)	1.715	0.196	0.058
		MAX	4.44 (6.18)	5.45 (4.55)	6.52 (6.35)	1.757	0.188	0.059
		MIN	−8.21 (4.04)	−7.04 (5.46)	−5.45 (5.34)	3.732	**0.03**	0.118
		ROM	12.66 (3.69)	12.49 (3.97)	11.97 (3.71)	0.33	0.707	0.012
Ankle	SP	IC	17.45 (6.73)	15.34 (4.72)	14.53 (6.02)	4.168	**0.021**	0.13
		MAX	29.11 (3.69)	27.71 (4.30)	27.11 (3.39)	3.323	**0.043**	0.106
		MIN	−8.47 (7.82)	−9.29 (4.37)	−8.98 (5.99)	0.13	0.878	0.005
		ROM	37.59 (5.78)	37.00 (3.64)	36.10 (5.67)	0.615	0.53	0.021
	CP	IC	2.95 (6.59)	2.70 (3.84)	1.54 (6.16)	0.53	0.591	0.018
		MAX	7.74 (5.05)	6.43 (2.89)	5.14 (5.74)	2.478	0.093	0.079
		MIN	−9.07 (4.89)	−9.68 (2.87)	−9.80 (4.48)	0.293	0.747	0.01
		ROM	16.81 (4.03)	16.11 (3.29)	14.95 (3.28)	2.214	0.118	0.071
NH		NH(mm)	10.92 (2.69)	12.48 (1.75)	12.93 (3.97)	23.653	**0.001**	0.449
VALR (BW/s)			53.14 (16.49)	58.84 (13.49)	64.18 (22.11)	3.775	**0.038**	0.095
GRF (BW)			2.01 (0.16)	2.02 (0.16)	2.08 (0.29)	2.018	0.149	0.046

*Note:* Bold *p*‐values indicate a statistically significant difference (*p* < 0.05).

Abbreviations: CP: coronal plane; GRF: Ground Reaction Force; HM: High midsole (52C); IC: Initial Contact; MM: Medium midsole (47C); NH: Navicular height; ROM: Range of Motion; SM: Soft midsole (42C); SP: sagittal plane; TP: transverse plane; VALR: Vertical average loading rate.

In contrast, for the peak vertical ground reaction force (vGRF), although the value increased slightly with increasing hardness (from 2.01 BW to 2.08 BW), statistical analysis indicated no significant difference among the three hardness conditions (*p >* 0.05).

### 3.4 Relative Contribution of Integrated Electromyography (iEMG)

**FIGURE 4:**
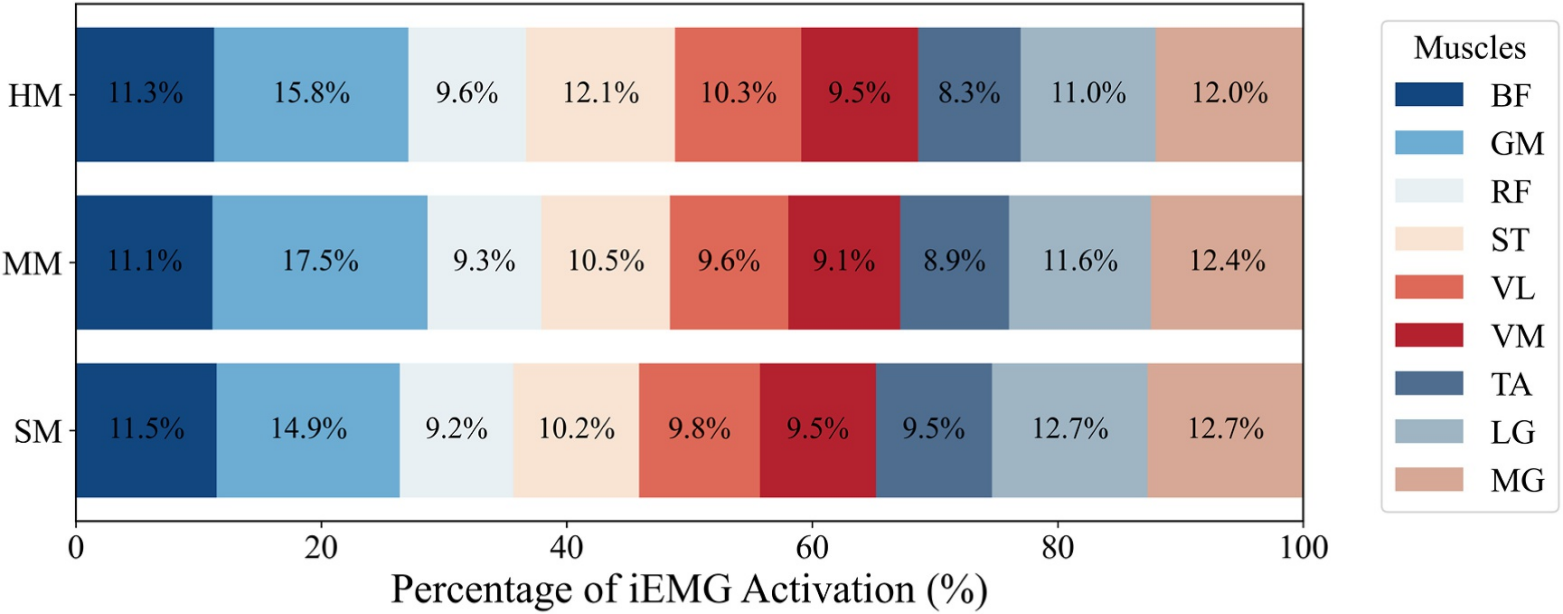
Proportion of iEMG for each muscle relative to total activation at three midsole hardness conditions. BF, biceps femoris; GM, gluteus maximus; LG, lateral gastrocnemius; MG, medial gastrocnemius; RF, rectus femoris; ST, semitendinosus; TA, tibialis anterior; VL, vastus lateralis; VM, vastus medialis.

Figure 4 shows the distribution of the relative contribution rates of iEMG signals from various muscle groups during the stance phase of running under three different MH conditions. As hardness increased, the relative contribution rate of the ST rose significantly (*p =* 0.011). Post hoc tests indicated that under the HM condition, the relative contribution of the ST reached 12.1%, which was significantly higher than the 10.2% observed under the SM condition (*p =* 0.026) and the 10.5% observed under the MM condition (*p =* 0.044). For the GM, the main effect of hardness was also significant (*p =* 0.002); the contribution rate under the MM condition was 17.5%, significantly higher than the 14.9% under the SM condition (*p =* 0.006) and the 15.8% under the HM condition (*p =* 0.028).

The relative contribution of the distal calf muscles showed a significant decreasing trend as hardness increased. For the TA, the main effect of hardness was significant (*p =* 0.046), with its contribution rate decreasing significantly from 9.5% under the SM condition to 8.3% under the HM condition (*p =* 0.025). Similarly, the relative contribution of the LG was significantly affected by hardness (*p =* 0.010), decreasing significantly from 12.7% under the SM condition to 11.0% under the HM condition (*p =* 0.024). For the other muscles tested, including BF, RF, VL, VM, and MG, although there were minor numerical fluctuations between the different hardness levels, statistical analysis did not reveal any significant differences (*p >* 0.05).

### 3.5 Average Muscle Activation

**FIGURE 5:**
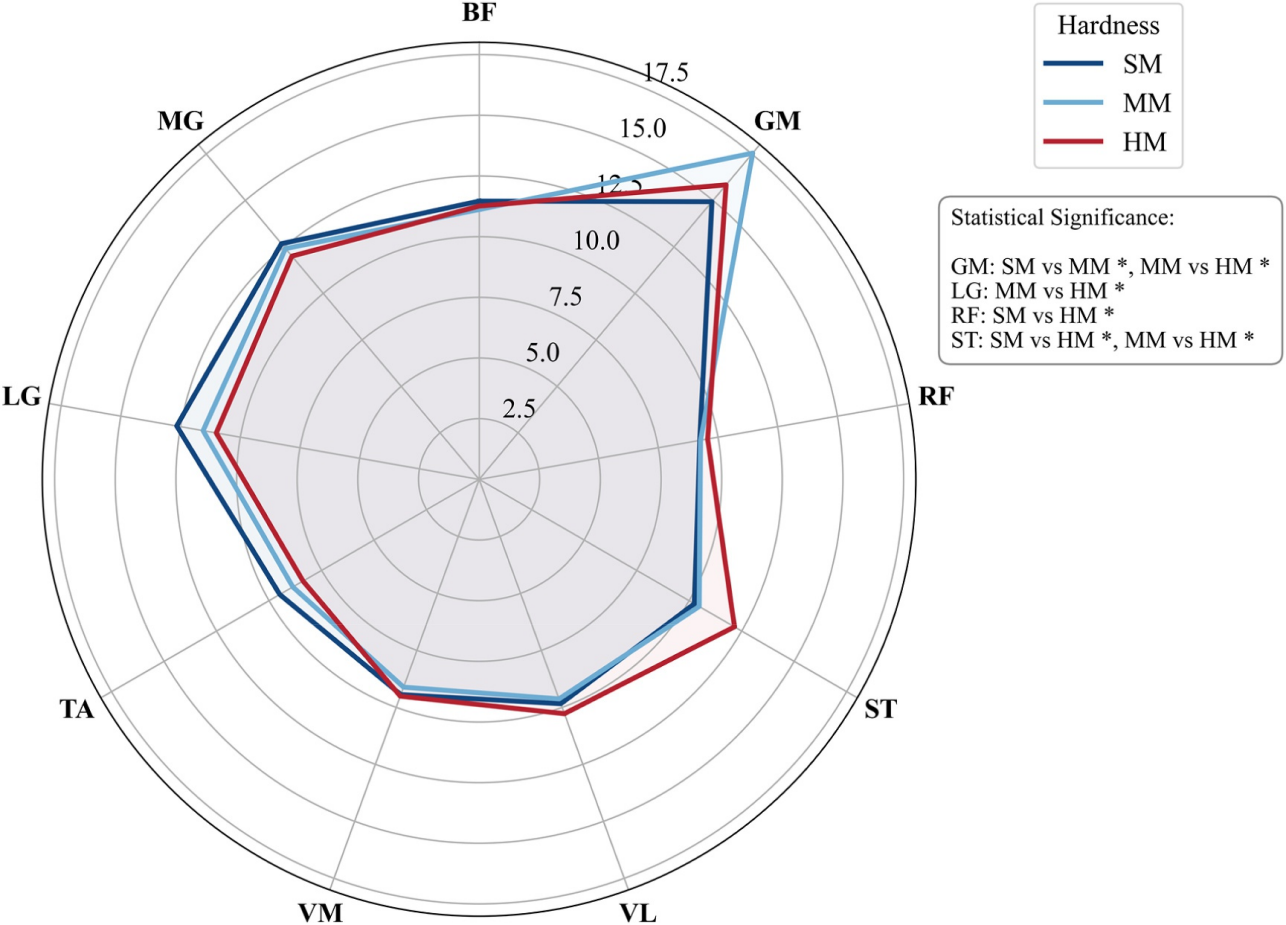
Radar chart illustrating the lower limb muscle activation patterns under three different midsole hardness conditions. BF, biceps femoris; GM, gluteus maximus; LG, lateral gastrocnemius; MG, medial gastrocnemius; RF, rectus femoris; ST, semitendinosus; TA, tibialis anterior; VL, vastus lateralis; VM, vastus medialis.

The activation level of the ST showed a significant upward trend as hardness increased (*p =* 0.011) (Figure 5). Post hoc tests indicated that the mean ST activation level under the HM condition (0.248) was significantly higher than that under the SM (0.201, *p =* 0.025) and MM (0.202, *p =* 0.043) conditions. Furthermore, for the GM, the main effect of hardness was also significant (*p =* 0.002), but it exhibited a different pattern of change: the activation level under the MM condition (0.38) reached a peak, significantly higher than that under the SM condition (0.31, *p =* 0.006) and the HM condition (0.32, *p =* 0.028).

The average activation level of the distal calf muscles showed a significant decreasing trend as hardness increased. For the TA, the main effect of hardness was significant (*p =* 0.048), with its activation level decreasing significantly from 0.194 under the SM condition to 0.171 under the HM condition (*p =* 0.026). Similarly, the activation level of the LG was significantly affected by hardness (*p =* 0.010), showing a significant decrease from the SM condition (0.259) to the HM condition (0.224) (*p =* 0.024).

## 4 Discussion

The present investigation was designed to examine how running shoe MH influences lower‐extremity biomechanics and muscle activation during the stance phase of pediatric running. The findings demonstrate that increasing MH was associated with changes in selected lower‐limb kinematic, impact loading, and muscle‐activation variables. Kinematically, increasing MH was accompanied by reductions in selected joint‐motion variables and a concomitant elevation in NH; simultaneously, kinetic analysis revealed a significant increase in VALR, which was generally consistent with the initial hypothesis [48]. At the muscular level, the decreased activation of the TA and LG together with the increased activation of the ST may reflect differences in muscular recruitment across MH conditions, including a possible shift in muscular involvement from distal to more proximal muscles. However, the absolute differences in joint motion and muscle activation among the three MH conditions were relatively small, and several measured variables showed no significant pairwise differences. Therefore, although MH elicited statistically detectable acute responses in selected variables, the magnitude and potential functional relevance of these differences should be interpreted cautiously.

The significantly lower NH observed under the SM condition indicates a lower navicular position and potentially greater arch deformation during stance [25]. This finding was accompanied by greater ankle sagittal‐plane motion, with both the maximum ankle sagittal angle and the ankle angle at IC being significantly greater under the SM condition than under the HM condition. In addition, hip sagittal‐plane ROM was significantly greater under the SM condition than under the HM condition. Together, these findings suggest that the softer midsole condition was associated with a lower navicular position and greater sagittal‐plane motion at the ankle and hip. The greater joint motion observed under the softer midsole condition may represent one component of the lower‐limb mechanical response to differences in footwear cushioning [6, 49, 50]. At the muscular level, the greater activation of the TA and LG under the SM condition indicates greater involvement of these distal muscles during stance. Previous footwear studies have also reported that changes in shoe mechanical properties can alter the activation of the tibialis anterior and gastrocnemius muscles [20, 51]. In the present study, these differences may reflect an adjustment in muscular recruitment associated with the altered foot–shoe mechanical condition rather than evidence of a specific neural‐control strategy. However, because dynamic stability, joint stiffness, and energy absorption were not directly measured, the functional consequences of these differences cannot be determined from the present data.

Under the HM condition, the significantly higher NH compared with the SM condition indicates a higher navicular position and potentially less arch deformation during stance. This finding was accompanied by smaller sagittal‐plane motion at the ankle and hip, with the maximum ankle sagittal angle, ankle angle at IC, and hip sagittal‐plane ROM being significantly lower under the HM condition than under the SM condition. These kinematic differences suggest that the harder midsole condition was associated with reduced sagittal‐plane lower‐limb motion. At the same time, VALR was significantly higher under the HM condition than under the SM condition, whereas peak vGRF did not differ significantly among the three conditions. This suggests that MH influenced the rate at which vertical force developed during early stance rather than the magnitude of the peak vertical force itself. This interpretation is supported by previous evidence showing that initial foot‐contact kinematics are associated with impact loading rate during running [52]. Importantly, these loading responses should be interpreted in the context of pediatric gait, as school‐age children are still undergoing musculoskeletal and neuromotor development; therefore, footwear‐related responses observed in adults may not be directly generalizable to children [13]. At the muscular level, the greater activation of the ST under the HM condition indicates increased involvement of this proximal muscle during stance. Together with the lower activation of the TA and LG, this pattern may be consistent with a possible redistribution of muscular involvement from distal to more proximal muscles as MH increases. However, because joint stiffness, energy absorption, muscle co‐contraction, and neural‐control strategies were not directly assessed, the mechanisms underlying these changes cannot be determined from the present data.

A notable finding of this study was that GM activation and relative contribution were highest under the MM condition. The GM plays an important role in hip control during running [53]. However, greater muscle activation alone does not necessarily indicate greater power output or more efficient propulsion. Within the three tested conditions, MM showed the highest GM activation and relative contribution, together with an intermediate mean VALR; however, the pairwise differences in VALR involving MM were not statistically significant. Therefore, these findings indicate a distinct response pattern under MM rather than evidence of a more favorable or balanced biomechanical condition. This cautious interpretation is consistent with the perspective of Santo et al., who suggested that footwear‐related changes in muscle activity should not necessarily be interpreted as additional functional benefits in healthy individuals [54]. However, because running economy, dynamic stability, joint power, propulsion performance, and comfort were not directly assessed, the present findings do not establish MM as an optimal midsole‐hardness condition or demonstrate additional functional benefits.

This study has several limitations. First, the experiments were conducted in a controlled indoor force‐plate environment, which may not fully represent the variable surface and environmental conditions encountered during children's habitual running. Second, the study population was limited to healthy boys within a specific age range; therefore, the findings should be generalized cautiously to girls or children at other developmental stages. Third, the present study examined only acute responses to different MH conditions. Therefore, the relatively small differences observed in several kinematic and muscle‐activation variables should not be interpreted as evidence of long‐term adaptation, injury prevention, or performance benefits. In addition, joint stiffness, joint moments and power, running economy, dynamic stability, comfort, muscle co‐contraction, muscle synergies, and neural‐control variables were not directly assessed. Consequently, the mechanisms underlying the observed changes in joint motion and muscle activation, as well as their functional significance, cannot be determined from the present data. Furthermore, surface EMG was limited to the selected superficial muscles and therefore did not characterize the contribution of deep muscles or complete intermuscular coordination. The study was also limited to a single running speed and did not include other movement tasks commonly performed by children, such as jumping or changing direction. Future studies should incorporate broader participant groups, multiple running speeds and movement tasks, and additional kinetic and neuromuscular measures to determine whether the acute responses observed here have meaningful functional or long‐term implications.

## 5 Conclusions

Running shoe MH influenced the way children responded biomechanically and muscularly during the stance phase of running. Softer midsoles were associated with a lower navicular position, greater sagittal‐plane motion at the ankle and hip, and greater involvement of the TA and LG, whereas harder midsoles were associated with reduced sagittal‐plane motion, a higher rate of vertical force development without a corresponding increase in peak vGRF, and greater ST involvement. The MM condition showed a different response pattern, characterized by greater GM involvement and an intermediate loading rate within the tested conditions. Overall, these findings suggest that changing MH modifies the distribution of foot–lower‐limb motion, impact loading characteristics, and muscular involvement, but the observed acute differences were relatively modest and do not indicate that any of the tested hardness conditions are functionally superior.

## Author Contributions

**Zhaolong Ye:** conceptualization, investigation, methodology, software, visualization, writing – original draft, formal analysis. **Bojie Xuan:** conceptualization, investigation, methodology, software, visualization, writing – original draft, writing – review and editing, formal analysis, supervision. **Fengping Li:** investigation, methodology, software, resources, writing – review and editing. **Kuiyu Chen:** investigation, methodology, software, resources, writing – review and editing. **Yiyao Chen:** investigation, methodology, software, resources, writing – review and editing. **Zhanyi Zhou:** investigation, methodology, software, resources, writing – review and editing. **Diwei Chen:** investigation, methodology, software, resources, writing – review and editing. **Dongxu Wang:** investigation, methodology, software, resources, writing – review and editing. **Xuanzhen Cen:** investigation, methodology, software, resources, writing – review and editing. **Yufei Li:** conceptualization, funding acquisition, investigation, methodology, resources, software, supervision, visualization, writing – review and editing. **Dong Sun:** investigation, methodology, software, resources, writing – review and editing. **Gusztáv Fekete:** investigation, methodology, software, resources, writing – review and editing. **Danica Janicijevic:** investigation, methodology, software, resources, writing – review and editing. **Yang Song:** conceptualization, methodology, visualization, writing – review and editing, supervision, funding acquisition. **Yaodong Gu:** conceptualization, methodology, visualization, writing – review and editing, supervision, funding acquisition. All authors contributed to drafting and critically revising the work. All authors read and approved the final version of the article.

## Funding

This study was sponsored by National Key R&D Program of China (2024YFC3607305), General Program of Guangdong Provincial Natural Science Foundation of China (2026A1515010084), Joint Fund of Zhejiang Provincial Natural Science Foundation of China (LKLY26H170001), Zhejiang Provincial Key Project of Education Science Planning (2025SB084), Scientific Research Fund of Zhejiang Provincial Education Department (Y202559510), Ningbo Key Research and Development Program (2022Z196), Zhejiang Rehabilitation Medical Association Scientific Research Special Fund (ZKKY2023001, ZKKY2024012), Ningbo Clinical Research Center for Orthopedics and Exercise Rehabilitation (No. 2024L004), Research Academy of Medicine Combining Sports, Ningbo (No. 2023001), Start‐up Research Funding from Ningbo University (ZX2026000474), and K. C. Wong Magna Fund in Ningbo University. China Postdoctoral Science Foundation (2024M751541).

## Ethics Statement

The experimental protocol was approved by the Scientific Ethics Committee of Ningbo University (Approval No.: TY2025065). All procedures were conducted in accordance with the ethical tenets of the Declaration of Helsinki. Written informed consent was obtained from all participants and their legal guardians prior to the start of the study.

## Consent

Written consent was obtained from all participants via consent form prior to any participation within the study protocol.

## Conflicts of Interest

Yufei Li is an employee of ANTA Sports Products Limited. The experimental footwear used in this study was provided by ANTA Sports Products Limited. The authors declare no conflicts of interest.

## Data Availability

The datasets used and/or analyzed for this study are available from the corresponding author upon reasonable request.
